# Comparison of the visual performance of iris-fixated phakic lens and implantable collamer lens to correct high myopia

**DOI:** 10.1186/s12886-021-01995-3

**Published:** 2021-06-02

**Authors:** Xiao-ling Jiao, Jun Li, Zhe Yu, Ping-hui Wei, Hui Song

**Affiliations:** Tianjin Eye Hospital, Tianjin Key Lab of Ophthalmology and Visual Science, Nankai University Affiliated Eye Hospital, Clinical College of Ophthalmology Tianjin Medical University, Tianjin Eye Institute, No. 4 Gansu Road, Heping District, Tianjin, 300020 China

**Keywords:** Phakic intraocular lens, Implantable collamer lenses, Light scattering, Aberration, Modulation transfer function

## Abstract

**Background:**

To compare visual performance between the iris-fixated phakic intraocular len (pIOL) and implantable collamer len (ICL) to correct high myopia.

**Methods:**

Twenty-four eyes underwent iris-fixated pIOL implantation and 24 eyes underwent ICL implantation. At the 6-month follow-up, the best-corrected visual acuity (BCVA) and uncorrected distance visual acuity (UDVA) were compared between the iris-fixated pIOL and ICL groups. The objective scatter index (OSI), modulation transfer function (MTF) cutoff, and ocular aberrations were performed to evaluate postoperative visual quality between the two groups.

**Results:**

No significant difference was found in UDVA, BCVA, and spherical equivalent between the iris-fixated pIOL and ICL groups (*P* > 0.05). Six months after surgery, the following values were significantly higher in the ICL group than in the iris-fixated pIOL group: MTF cutoff, strehl ratio and optical quality analysis system values at contrasts of 9 %, 20 %, and 100 % (*P* < 0.01). The OSI in the iris-fixated pIOL group was higher than in the ICL group 6 months after surgery (*P* < 0.01). All high-order aberrations were slightly more severe in the iris-fixated pIOL group than in the ICL group 6 months after surgery, although only trefoil (*P* = 0.023) differed significantly in this regard.

**Conclusions:**

Both iris-fixated lenses and ICLs can provide good visual acuity. ICLs confer better visual performance in MTF-associated parameters and induce less intraocular light scattering than iris-fixated pIOLs.

## Background

Myopia is a significant public health issue in China [1]. Phakic intraocular len (pIOL) implantation and corneal laser refractive surgery are widely used to correct myopia, but small-incision lenticule extraction, laser-assisted *in situ* keratomileusis, and some other corneal laser surgeries are too risky in patients with severe myopia because of the cornea’s biomechanical limits [2]. However, pIOL implantation can be used regardless of corneal thickness and topography, and thus is more suitable in such patients. Moreover, recent studies have shown that pIOL implantation is efficacious and safe in low-to-moderate myopia, as well as in early keratoconus [3, 4]. Therefore, pIOL implantation is increasing in popularity.

According to the site of implantation, pIOLs are classified as iris-fixated pIOLs, posterior chamber lenses, and anterior chamber angle-supported lenses. However angle-supported pIOLs are associated with corneal endothelial cell loss, iris retraction, secondary glaucoma, and subsequent pupil ovalization and are rarely used [5, 6], while iris-fixated pIOLs and posterior chamber lenses are widely used and beneficial in high myopia [7, 8].

Previous studies have reported that implantation of iris-fixated pIOLs and implantable collamer lenses (ICLs) is safe, efficient, predictable, and stable [9, 10]. Iris-fixated pIOLs are always properly centered over the pupil, conferring stable vision. However, the rigid iris-fixated pIOLs require a larger incision in the sclera, which can increase astigmatism in the immediate postoperative period. Furthermore, peripheral iridectomy increases the risk associated with pIOL implantation, and the hole in the iris might cause light scattering. While the latest generation of posterior chamber ICL has an additional 360 μm central hole that can eliminate the need for peripheral iridectomy. They also increase aqueous humor circulation, nourishing the lens [11]. There are several studies compared the visual performance of iris-fixated pIOLs and ICLs [12–14]. But the ICL without a central hole was evaluated in these studies. At present, the ICL with a central hole was widely used in clinic. But it remains unclear whether the central hole affects vision quality, aberration, and MTF profile of eye. In ICLs, the diameter of the optical zone changes from 4.5 to 5.8 mm. This limited size may lead to glare and halo, so subjective and objective vision quality must be further compared between iris-fixated pIOLs and ICLs with a central hole in the correction of high myopia.

Intraocular light scattering is an indicator of visual performance after surgery [15]. Increased intraocular light scattering in pseudophakic eyes can result in glare, halos. After iris-fixated pIOL implantation, peripheral iridectomy may induce more intraocular light scattering, as may the central hole in the ICL. The present study aimed to determine which types of pIOL induce less intraocular light scattering and to compare visual performance between iris-fixated pIOLs and ICLs.

## Methods

### Subjects and methods

The present retrospective study included 24 eyes of 12 patients (8 women, 4 men) implanted with an iris-fixated pIOL (Verisyse; Abbott Medical Optics, Santa Ana, USA) (iris-fixated pIOL group) between January 2011 and March 2015 and 24 eyes of 12 patients (7 women, 5 men) implanted with an ICL (Visian ICL v4c; STAAR Surgical, Nidau, Switzerland) (ICL group) between September 2018 and November 2020. All subjects were fully informed about the possible complications and the informed consents were obtained. All procedures were in accordance with the tenets of the Declaration of Helsinki, and the study protocol was approved by the local ethics committee (Tianjin Eye Hospital Ethics Committee).

The inclusion criteria were as follows: age ranged from 18 to 45 years, refractive error remaining stable for at least 2 years (change of < 0.5 D), anterior chamber depth ≥ 3.0 mm, high myopia ≥ 6 D, astigmatism < 1.0 D in both groups, endothelial cell density > 2200 cells/mm^2^, intraocular pressure (IOP) < 21 mmHg. The exclusion criteria were as follows: history of ocular surgery, ocular disease, such as retinal detachment, maculopathy, retinopathy, glaucoma, corneal opacities, or ocular inflammation.

### Surgical technique

A Verisyse IOL (Verisyse; Abbott Medical Optics, Santa Ana, USA) was used in iris-fixated pIOL group. The iris-fixated pIOL power was calculated by using SRK/T formula and aimed to emmetropia. A 6.0-mm corneoscleral limbus incision was performed at the 11:30 o’clock position. Two 1.0-mm paracenteses were performed at the 2 and 10 o’clock positions. Carbamylcholine chloride (0.1 mL; Furuida Co., Shandong, China) was then injected into the anterior chamber. The iris-fixated pIOL was inserted into the anterior chamber and fixed to the iris using a special holder. The lens was enclavated between 3 and 9 o’clock, so that the haptics were oriented at the 3 and 9 o’clock positions. Peripheral iridectomy was performed at 12 o’clock to avoid pupillary block glaucoma. The viscoelastic substance was removed and the primary incision was closed using three interrupted 10 − 0 non-absorbable nylon sutures. Antibiotic and anti-inflammatory eye drops were administered and reduced gradually for 1 month. The corneal sutures were removed 3–4 weeks after surgery.

An ICL with a central hole (Visian ICL v4c; STAAR Surgical, Nidau, Switzerland) was implanted in ICL group. The size and power of ICL was calculated with formula provided by the manufacturer. The white-to-white and the anterior chamber depth measured by Pentacam system (Oculus; Wetzlar, Germany) codetermined the ICL size. Before surgery, topical 0.5 % tropicamide and 0.5 % phenylephrine eye drops (Mydrin-P; Santen Pharmaceutical) were used to sufficiently dilate the pupil. Proparacaine (Ruinian Best Pharmaceutical, Nanjing) was used to induce topical anesthesia. A 3.0-mm temporal corneal incision was then made. The V4c ICL was inserted into the anterior chamber through the corneal incision, and positioned into the ciliary sulcus. The viscoelastic substance was removed and the primary incision was hydrated. Antibiotic and anti-inflammatory eye drops were administered and reduced gradually for 1 month.

### Ophthalmologic measurements

Ophthalmologic measurements were recorded immediately before and 6 months after surgery. The uncorrected distance visual acuity (UDVA), manifest refraction, best-corrected visual acuity (BCVA), endothelial cell count (ECC; Topcon SP-2000P; Topcon, Tokyo, Japan), IOP (TX-10 non-contact tonometer; Canon, Japan), axial length, and anterior chamber depth (IOLMaster; Carl Zeiss Meditec AG, Germany) were measured. At a pupil diameter of 4.0 mm and 6.0 mm, the spherical aberration, coma, trefoil, and root mean square of the total eye were measured using iTrace (Tracey Technology, Houston, TX, USA). The optical quality analysis system (OQAS) values at contrasts of 100 %, 20 %, and 9 % (OV-100, OV-20, and OV-9), modulation transfer function (MTF) cutoff, objective scatter index (OSI), and strehl ratio were measured using an OQAS (Visiometrics, Tarrasa, Spain). This system automatically corrects spherical refractive error between − 3 and + 3 D. Astigmatism and spherical refractive error of more than ± 3 D were corrected by placing an appropriate cylindrical or spherical lens in front of the eye. The parameters measured by the OQAS were under a 4.0-mm artificial pupil. Topical 0.5 % phenylephrine and 0.5 % tropicamide eye drops (Mydrin-P; Santen Pharmaceutical) were used to dilate the pupils before the iTrace and OQAS examinations, and at least 3 measurements were obtained. The entire ocular aberration was measured in the right eye of each subject with pupil diameter of 4.0 and 6.0 mm .

### Statistical analysis

Data were presented as mean ± standard deviation and analyzed using SPSS for Windows 19.0 software (SPSS, Chicago, IL, USA). Snellen visual acuity was changed to the logarithm of the minimum angle of resolution (logMAR). The Shapiro–Wilk test was used to confirm the normality of data distribution. The generalized linear model was used to analyze the bilateral data for avoiding the bias caused by inter-eye correlation. For other data, the Student’s t-test and Mann–Whitney U test were used to analyze the parametric and non-parametric data, respectively. *P* values < 0.05 were considered significant.

## Results

### Follow-up and baseline comparisons

Table 1 shows the patients’ demographics. All were followed up for 6 months. No significant differences were found in mean spherical equivalent (SE), UDVA, or BCVA between two groups either before or 6 months after surgery (*P* > 0.05; Table 1). No intraoperative or severe postoperative complications occurred in any patient, such as posterior capsule rupture, hyphema, serious anterior chamber inflammation, damage to the crystalline lens or cornea, macular edema, or retinal detachment. Corneal edema was observed in four eyes after iris-fixated pIOL implantation; it disappeared 3 days after surgery. High IOP (≤ 30 mmHg) was observed in six eyes after ICL implantation. Carteolol hydrochloride eye drops (China Otsuka Pharmaceutical, Tianjin, China) were prescribed twice per day, and the IOP of all six eyes recovered within 5 days.

**Table 1:** Preoperative data and postoperative data in both groups

Characteristic	Iris-fixated pIOLs (*n* = 24)	ICL (*n* = 24)	*P* value
Age (years)	25.25 ± 1.76	25.58 ± 1.73	0.645
Gender (male : female)	4:8	5:7	0.673
Preop.			
UDVA (LogMAR)	1.22 ± 0.48	1.14 ± 0.47	0.338
BCVA (LogMAR)	0.03 ± 0.04	0.02 ± 0.04	0.677
Mean sphere (D)	-12.84 ± 1.87	-12.96 ± 2.11	0.844
Mean cylinder (D)	-0.06 ± 0.83	-0.23 ± 0.81	0.469
Mean SE (D)	-12.95 ± 1.75	-12.98 ± 2.18	0.956
Axial length (mm)	28.15 ± 1.44	28.17 ± 1.57	0.971
Anterior chamber depth (mm)	3.43 ± 0.11	3.41 ± 0.10	0.554
Postop.			
UDVA (LogMAR)	-0.09 ± 0.08	-0.05 ± 0.05	0.949
BCVA (LogMAR)	-0.05 ± 0.07	-0.33 ± 0.06	0.470
Mean sphere (D)	-0.21 ± 0.23	-0.14 ± 0.27	0.089
Mean cylinder (D)	-0.14 ± 0.79	-0.05 ± 0.81	0.421
Mean SE (D)	-0.28 ± 0.37	-0.11 ± 0.47	0.178

*UDVA* Uncorrected distant visual acuity, *BCVA* Best corrected distant visual acuity, *SE* spherical equivalent

**Fig. 1:**
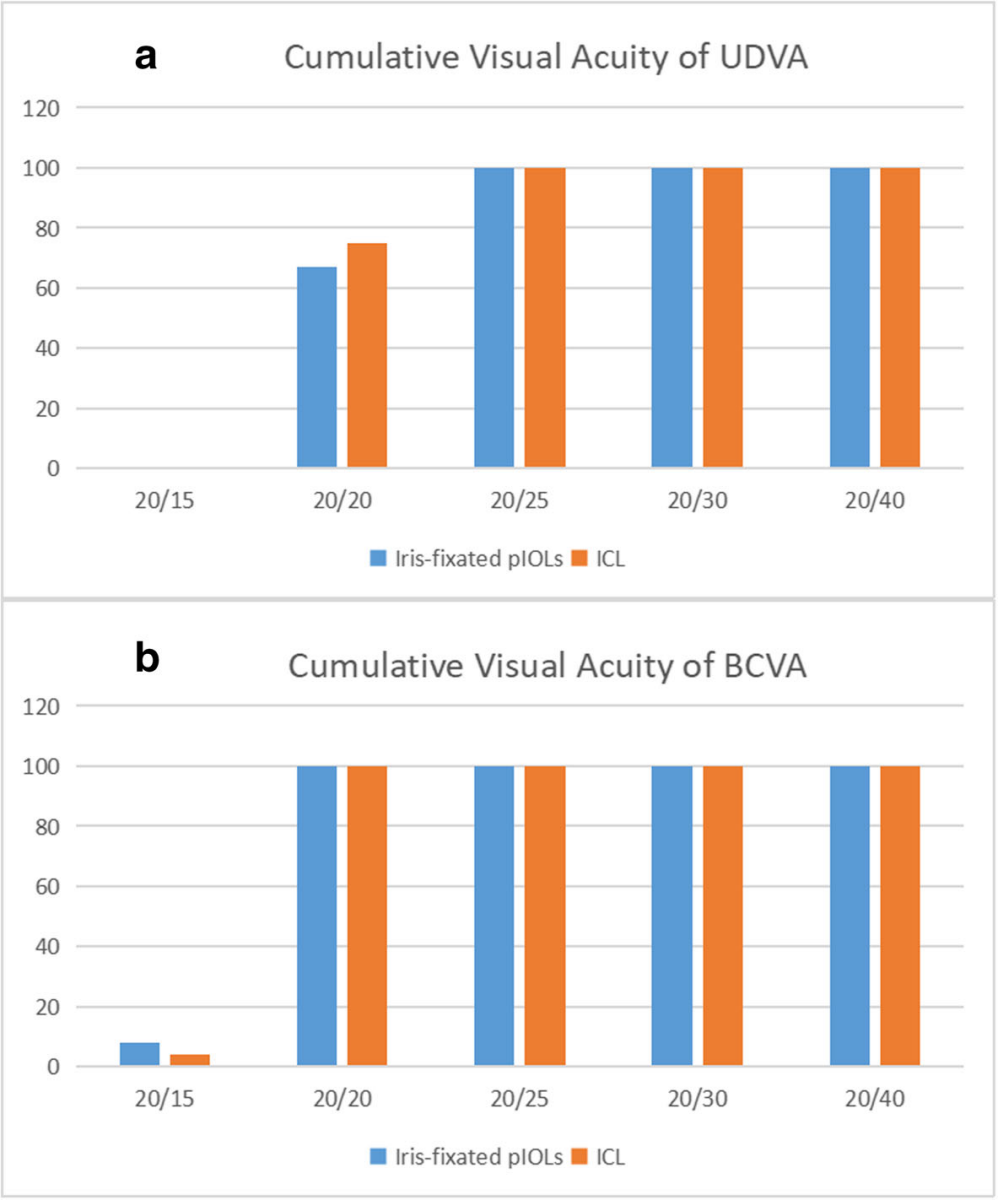
Cumulative visual acuity 6 months postoperatively in iris-fixated pIOL group and ICL group. **a.** uncorrected distance visual acuity (UDVA); **b.** best-corrected visual acuity (BCVA)

Six months after surgery, the UCVA was 20/20 or better in 16 of 24 eyes (67 %) in the iris-fixated pIOL group, as well as in 18 of 24 eyes (75 %) in the ICL group (Fig. 1a). Meanwhile, the BCVA was 20/20 or better in 24 of 24 eyes (100 %) in the iris-fixated pIOL group, as well as in 24 of 24 eyes (100 %) in the ICL group (Fig. 1b).

**Fig. 2:**
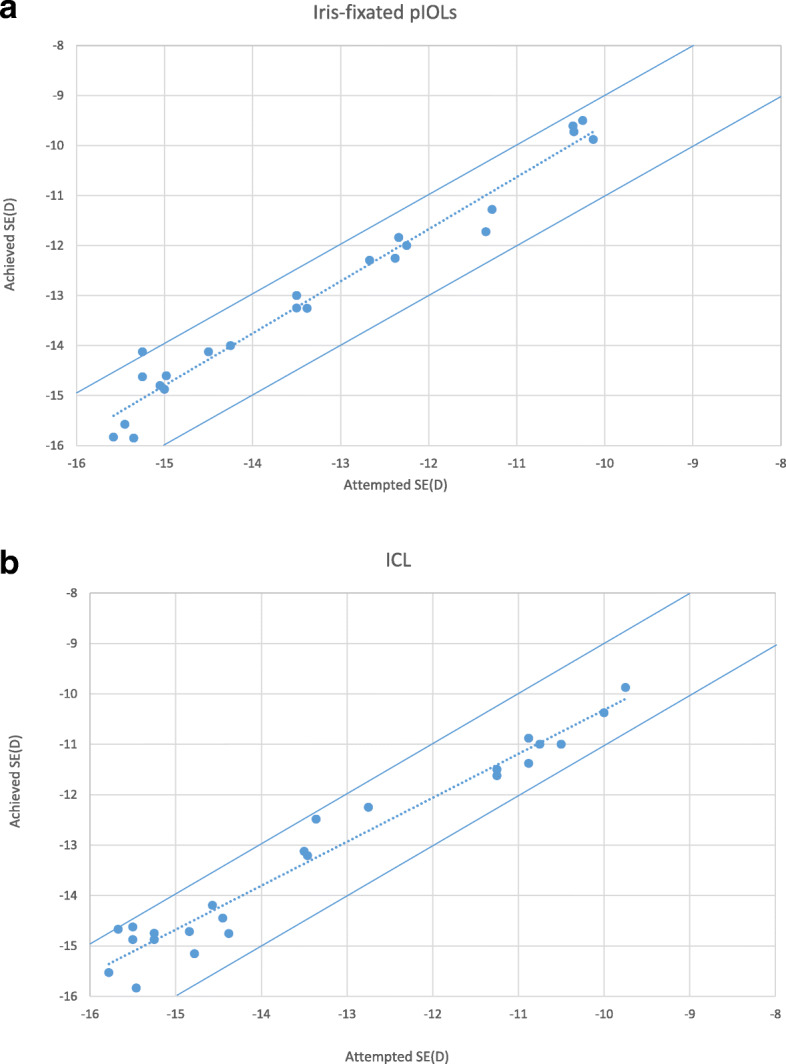
Plot of achieved vs. attempted correction for spherical equivalent. **a.** iris-fixated pIOL group; **b.** ICL group. The dashed lines in the plots represent the equality lines achieved SE = attempted SE, while the two solid lines that are parallel to the dashed lines are defined by ± 1 D

Figure 2 shows the achieved vs. attempted SE correction in both groups 6 months after surgery. When the SE was evaluated in the iris-fixated pIOL group at 6 months, 79 % of the eyes were within ± 0.50 D of emmetropia, while 96 % were within ± 1.00 D. Meanwhile, in the ICL group, 83 % of eyes were within ± 0.50 D of emmetropia, and 100 % were within ± 1.00 D.

### Corneal endothelial cell loss

Table 2 shows the endothelial cell loss in both groups. The rate of endothelial cell density loss did not differ significantly between the iris-fixated pIOL and ICL groups 6 months after implantation surgery.

**Table 2:** Corneal endothelial cell density and optical quality parameters six months after surgery

	Iris-fixated pIOLs	ICL	*P* value
Preop. ECD (cells/mm^2^)	3012 ± 113	3020 ± 135	0.579
Postop. ECD (cells/mm^2^)	2888 ± 131	2866 ± 127	0.223
ECD loss rate(%)	4.44 ± 2.44	5.03 ± 3.51	0.110
MTF cutoff frequency (cycle/degree)	21.34 ± 10.23	31.86 ± 10.81	< 0.001^#^
Strehl ratio	0.13 ± 0.06	0.16 ± 0.05	0.007^#^
OV 100 %	0.72 ± 0.34	0.98 ± 0.37	< 0.001^#^
OV 20 %	0.49 ± 0.27	0.67 ± 0.30	< 0.001^#^
OV 9 %	0.22 ± 0.10	0.46 ± 0.18	< 0.001^#^
OSI	2.77 ± 1.68	1.69 ± 1.11	< 0.001^#^

*ECD *Endothelial cell density, *MTF *modulation transfer function, *OSI *objective scattering index, *OV *Optical Quality Analysis System (OQAS) value; * = *P *< 0.05; # = *P *< 0.01

### Visual quality comparison

MTF is the contrast ratio between the retinal image and the original scene, and the MTF cutoff is the spatial frequency at 1 % of the maximum MTF. OV-9 and OV-20 are 1 and 5 % of the MTF, respectively. OV-100 is defined as the MTF cutoff frequency which is divided by 30 cycles per degree [16]. Comparison of aberration between iris-fixated pIOLs and ICLs was performed with a pupil diameter of 4 mm and 6 mm. MTF cutoff, OQAS values, and strehl ratio were lower in the iris-fixated pIOL group than in the ICL group 6 months after surgery, while the OSI values were higher (Table 2). There were significant differences in OV-100, OV-20, OV-9, MTF cutoff, Strehl ratio and OSI between the two groups (*P* < 0.05).

### Aberration comparison

Almost all aberrations were slightly more severe in the iris-fixated pIOL group than in the ICL group 6 months after surgery, specifically total aberrations, total low-order aberrations (tLOAs), total high-order aberrations (tHOAs), defocus, spherical aberrations, astigmatism, trefoil, and coma, although the difference was only significant for trefoil (*P* < 0.05) (Table 3).

**Table 3:** Aberration parameters in both groups

Internal aberrations	Pupil diameter	Iris-fixated pIOLs	ICL	*P*_2_ value
TA/D	4mm	1.08±.074	0.64±0.34	0.070
	6mm	2.23±1.5	1.47±0.89	0.147
	*P*_1_ value	0.026^*^	0.006^#^	
tLOAs/D	4mm	0.88±0.60	0.54±0.35	0.101
	6mm	1.5±0.91	1.13±0.61	0.243
	*P*_1_ value	0.059	0.008^#^	
Defocus/D	4mm	0.62±0.58	0.34±0.27	0.151
	6mm	0.76±0.57	0.72±0.58	0.853
	*P*_1_ value	0.549	0.058	
Astigmatism/D	4mm	0.51±0.4	0.25±0.24	0.066
	6mm	1.93±2.97	0.73±0.51	0.182
	*P*_1_ value	0.115	0.008^#^	
tHOAs/D	4mm	0.40±0.41	0.23±0.24	0.237
	6mm	1.52±1.41	0.82±0.76	0.144
	*P*_1_ value	0.015^*^	0.019^*^	
Coma/D	4mm	0.37±0.52	0.1±0.11	0.098
	6mm	0.58±0.47	0.44±0.54	0.507
	*P*_1_ value	0.322	0.048^*^	
Spherical/D	4mm	0.17±0.30	0.05±0.1	0.212
	6mm	0.15±0.30	0.24±0.33	0.500
	*P*_1_ value	0.867	0.080	
Trefoil/D	4mm	0.29±0.30	0.08±0.05	0.023^*^
	6mm	0.83±0.97	0.27±0.21	0.062
	*P*_1_ value	0.079	0.008^#^	

*P*_1_ = *P* value for 4mm and 6mm comparison in the same group; *P*_2_ = *P* value for comparison between iris-fixated pIOL group and ICL group

*TA* total aberration, *tHOAs* total high-order aberration, *tLOAs* total low-order aberration

^*^ = *P* < 0.05; ^#^ = *P* < 0.01

## Discussion

Previous studies have shown that implantation of iris-fixated pIOL and ICL is efficacious and safe for correcting high myopia [9, 16], and one meta-analysis showed no statistic difference in efficacy or safety between the two pIOLs [17]. In the present study, both iris-fixated pIOLs and ICLs markedly improved UCVA and BCVA, and no significant difference was found between the two, which was consistent with Awadein et al’s study [18].

Although rigid iris-fixated pIOLs require larger incision sizes, refractive results in the iris-fixated pIOL group were similar to those in the ICL group 6 months after surgery in the present study. In both groups, the postoperative SE was close to zero, with approximately 95 % of eyes within ± 1.00 D of emmetropia. The different incision sizes seemed not to affect postoperative astigmatism in iris-fixated pIOL group. Our results were consistent with those of Tahzib et al. [19], who compared refractive data after implantation of iris-fixated rigid and foldable pIOLs (Artiflex), finding that postoperative refractive astigmatism only differed significantly between groups after 1 week of follow-up, and that it was comparable after suture removal. Moreover, Coullet et al. [20] found no significant difference in postoperative astigmatism between foldable and rigid iris-fixated pIOLs beyond 3 months after surgery. In both studies, the corneal incision was closed with five or six interrupted sutures, which may have markedly decreased surgically induced astigmatism. In our study, the corneal limbus incision was closed using three interrupted sutures after iris-fixated pIOL implantation, and the sutures were removed 3–4 weeks after surgery.

The present research revealed that the values of MTF cutoff, strehl ratio, OV-100, OV-20, and OV-9 in the ICL group were significantly higher than in the iris-fixated pIOL group. Nochez et al. [21] showed that trefoil was associated with objective contrast sensitivity. In the present study, the postoperative trefoil value in the ICL group was markedly lower than in the iris-fixated pIOL group with the pupil diameter of 4.0 mm. With the pupil diameter of 6.0 mm, the postoperative trefoil value in the ICL group was lower than iris-fixated pIOL group, but there was no significant difference between the two groups. Perhaps the postoperative trefoil affected the MTF associated parameters. The relationship between MTF and higher-order aberrations requires further study.

The OSI, measured using OQAS, quantifies intraocular scattered light. Lower OSI corresponds to better optical quality [22]. The OSI was significantly higher in the iris-fixated pIOL group than in the ICL group after surgery in the present study. The ICL was placed into the ciliary sulcus behind the pupil, while the iris-fixated pIOL was enclavated to the iris in front of the pupil. In iris-fixated pIOL group, light leakage caused by peripheral iridectomy may increase intraocular scattered light, and iris-fixated pIOLs may cause greater trauma to the iris, manifesting as iris atrophy and pigment spread, both of which are irreversible and can affect direction of light. Our previous study has shown the ICL with a central hole doesn’t increase the intraocular light scattering [23]. Qin et al. [24] confirmed that ICLs induce less intraocular light scattering because they are rarely tilted or decentralized and their optical zone thickness is only 50–60 μm. As we know, the pupil diameter may be greater than 4.0 mm at night. The results of intraocular light scattering in this study can’t reflect the situation at night. However, further studies are needed to clarify this.

Alio and associates [12] compared intraocular aberrations in eight kind of phakic intraocular lens implantation by KR-1w, and discussed the impact on visual acuity that could appear after pIOL implantation. We used an iTrace visual function analyzer to evaluate HOAs after surgery and thus compare visual performance between iris-fixated pIOLs and ICLs. Alio et al. and Awadein et al. [12, 18] found no significant difference in HOAs and astigmatism between iris-fixated pIOLs and posterior chamber pIOLs at 4.0 mm and 6.0 mm pupil. Our results were in accordance with previous studies.

Alio et al. [12] found the values of postoperative coma in ICL group were significantly lower than iris-fixated pIOL group. In our study, the postoperative coma in ICL group was lower than iris-fixated pIOL group with a pupil diameter of 4.0mm and 6.0 mm, but no significant difference was observed between two groups. The aberration results were also consistent with Awadein et al.’s study [18]. The IOL decentration and tilt usually are the main reason to increase coma after IOL implantation [25]. The ICL was positioned in the ciliary sulcus, while the iris-fixated pIOL was enclavated to the iris. The difference of iris tissue enclavated by the bilateral Verisyse claws may lead to the decentration and tilt of the pIOL optic, which may result in the higher postoperative coma.

Our results showed a difference in ocular aberrations between the pupil diameter of 4.0 mm and 6.0 mm in ICL group. The small pupil diameter can lead to small HOAs. And ocular aberrations are known to increase with pupil diameter [26, 27]. Wang et al.’s [28] study also observed the coma-like aberration, spherical aberration, and other HOAs increased with the pupil size.

Karimian et al. [13] found the high order aberrations and spherical aberration were higher in iris-fixated than ICL group. But in our study no significant difference was observed in HOAs and spherical aberration between two pIOLs. Two reasons may result in this discrepancy. First, the mean age of Artiflex group was 30, while the mean age of ICL group was 27 in Karimian et al.’s study. The corneal HOAs and spherical aberration increases with age [26], which may result in the difference between the two groups; Second, aspherical IOL implantation generates fewer positive spherical aberrations than spherical IOL implantation after cataract surgery, leading to better contrast sensitivity and visual acuity [26]. Neither iris-fixated pIOLs nor ICLs have aspherical optic designs, which may be why there was no significant difference in spherical aberration between two groups in the present study.

In our study the postoperative trefoil in iris-fixated pIOL group was higher than ICL group. In the present study, a 6.0-mm corneoscleral limbus incision was performed in iris-fixated pIOL group, while a 3.0-mm temporal corneal incision was performed in ICL group. The larger corneal incision can increase the trefoil [29]. Tong et al. [30] also found the value of postoperative trefoil is dependent on incision size. The larger incision in rigid iris-fixated pIOL group may lead to higher postoperative trefoil in iris-fixated pIOL group.

Our study had several limitations. The follow-up time was insufficient and the sample size was small. Further study on big sample is needed. A previous study showed that visual acuity and refractive power become stable 1 month after ICL implantation surgery [31]. We compared clinical outcomes between groups 6 months after surgery, but long-term follow-up is necessary in future studies. However, further study is needed to confirm this conjecture.

## Conclusions

In conclusion, iris-fixated pIOLs and ICLs can provide good UCVA. ICL shows better visual performance than iris-fixated pIOL in MTF-associated parameters and induce less intraocular light scattering.

## Data Availability

All the data used in this study are available from the corresponding author.

## Abbreviations

pIOL: Phakic intraocular len
ICL: Implantable collamer len
BCVA: Best-corrected visual acuity
UDVA: Uncorrected distance visual acuity
OSI: Objective scatter index
MTF cutoff: Modulation transfer function cutoff
OV-100: Optical quality analysis system value at the contrast of 100 %
OV-20: Optical quality analysis system value at the contrast of 20 %
OV-9: Optical quality analysis system value at the contrast of 9 %
SE: Spherical equivalent

## References

[1] Li Y, Liu J, Qi P (2017). The increasing prevalence of myopia in junior high school students in the Haidian District of Beijing, China: a 10-year population-based survey. BMC Ophthalmol.

[2] Seiler T, Koufala K, Richter G (1998). Iatrogenic keratectasia after laser in situ keratomileusis. J Refract Surg.

[3] Kamiya K, Shimizu K, Igarashi A, Kitazawa Y, Kojima T, Nakamura T, Oka Y, Matsumoto R (2018). Posterior chamber phakic intraocular lens implantation: comparative, multicentre study in 351 eyes with low-to-moderate or high myopia. Br J Ophthalmol.

[4] Alió JL, Peña-García P, Abdulla GF, Zein G, Abu-Mustafa SK (2014). Comparison of iris-claw and posterior chamber collagen copolymer phakic intraocular lenses in keratoconus. J Cataract Refract Surg.

[5] Ardjomand N, Kölli H, Vidic B, El-Shabrawi Y, Faulborn J (2002). Pupillary block after phakic anterior chamber intraocular lens implantation. J Cataract Refract Surg.

[6] Kohnen T (2000). Searching for the perfect phakic intraocular lens. J Cataract Refract Surg.

[7] Güell JL, Morral M, Gris O, Gaytan J, Sisquella M, Manero F (2008). Five-year follow-up of 399 phakic Artisan-Verisyse implantation for myopia, hyperopia, and/or astigmatism. Ophthalmology.

[8] Kojima T, Kitazawa Y, Nakamura T, Takahashi M, Kamiya K, Ichikawa K, Igarashi A, Shimizu K (2018). Prospective Randomized Multicenter Comparison of the Clinical Outcomes of V4c and V5 Implantable Collamer Lenses: A Contralateral Eye Study. J Ophthalmol.

[9] van Rijn GA, Gaurisankar ZS, Ilgenfritz AP, Lima JEE, Haasnoot GW, Beenakker JM, Cheng YYY, Luyten GPM (2020). Middle- and long-term results after iris-fixated phakic intraocular lens implantation in myopic and hyperopic patients: a meta-analysis. J Cataract Refract Surg.

[10] Packer M (2018). The Implantable Collamer Lens with a central port: review of the literature. Clin Ophthalmol.

[11] Langeslag MJ, van der Mooren M, Beiko GH, Piers PA (2014). Impact of intraocular lens material and design on light scatter: In vitro study. J Cataract Refract Surg.

[12] Alio JL, Peña-García P, Pachkoria K, Alio JL 2nd, Aswad E. A. Intraocular optical quality of phakic intraocular lenses: comparison of angle-supported, iris-fixated, and posterior chamber lenses. Am J Ophthalmol. 2013;156:789–99. 10.1016/j.ajo.2013.05.013 23849312

[13] Karimian F, Baradaran-Rafii A, Hashemian SJ, Hashemloo A, Jafari ME, Yaseri M, Ghahari E, Akbarian S (2014). Comparison of three phakic intraocular lenses for correction of myopia. J  Ophthal Vis Res.

[14] Lee SY, Kwon HJ, Ahn HS, Seo KY, Kim EK, Kim TI (2011). Comparison of patient outcomes after implantation of Visian toric implantable collamer lens and iris-fixated toric phakic intraocular lens. Eye.

[15] Rizk IM, Al-Hessy AA, El-Khouly SE, Sewelam AM (2019). Visual performance after implantation of two types of phakic foldable intraocular lenses for correction of high myopia. Int J Ophthalmol.

[16] He T, Zhu Y, Zhou J (2020). Optical quality after posterior chamber Phakic implantation of an intraocular Lens with a central hole (V4c implantable Collamer Lens) under different lighting conditions. BMC Ophthalmol.

[17] Liang GL, Wu J, Shi JT, Liu J, He FY, Xu W (2014). Implantable collamer lens versus iris-fixed phakic intraocular lens implantation to correct myopia: a meta-analysis. PloS one.

[18] Awadein A, Habib AE (2013). ICL versus Veriflex phakic IOL for treatment of moderately high myopia: randomized paired-eye comparison. J Refract Surg.

[19] Tahzib NG, MacRae SM, Yoon G, Berendschot TT, Eggink FA, Hendrikse F, Nuijts RM (2008). Higher-order aberrations after implantation of iris-fixated rigid or foldable phakic intraocular lenses. J Cataract Refract Surg.

[20] Coullet J, Guëll JL, Fournié P, Grandjean H, Gaytan J, Arné JL, Malecaze F (2006). Iris-supported phakic lenses (rigid vs foldable version) for treating moderately high myopia: randomized paired eye comparison. Am J Ophthalmol.

[21] Nochez Y, Majzoub S, Pisella PJ (2012). Effect of interaction of macroaberrations and scattered light on objective quality of vision in pseudophakic eyes with aspheric monofocal intraocular lenses. J Cataract Refract Surg.

[22] Xu CC, Xue T, Wang QM, Zhou YN, Huang JH, Yu AY (2015). Repeatability and reproducibility of a double-pass optical quality analysis device. PloS one.

[23] Yu Z, Li J, Song H (2020). Short-time evaluation on intraocular scattering after implantable collamer lens implantation for correcting high myopia. BMC Ophthalmol.

[24] Qin Q, Bao L, Yang L, He Z, Huang Z (2019). Comparison of visual quality after EVO-ICL implantation and SMILE to select the appropriate surgical method for high myopia. BMC Ophthalmol.

[25] Lawu T, Mukai K, Matsushima H, Senoo T (2019). Effects of decentration and tilt on the optical performance of 6 aspheric intraocular lens designs in a model eye. J Cataract Refract Surg.

[26] Awwad ST, El-Kateb M, McCulley JP (2006). Comparative higher-order aberration measurement of the LADARWave and Visx WaveScan aberrometers at varying pupil sizes and after pharmacologic dilation and cycloplegia. J Cataract Refract Surg.

[27] McKelvie J, McArdle B, McGhee C (2011). The influence of tilt, decentration, and pupil size on the higher-order aberration profile of aspheric intraocular lenses. Ophthalmology.

[28] Wang Y, Zhao K, Jin Y, Niu Y, Zuo T (2003). Changes of higher order aberration with various pupil sizes in the myopic eye. J Refract Surg.

[29] Can İ, Bayhan HA, Çelik H, Ceran BB (2012). Comparison of corneal aberrations after biaxial microincision and microcoaxial cataract surgeries: a prospective study. Curr Eye Res.

[30] Tong N, He JC, Lu F, Wang Q, Qu J, Zhao YE (2008). Changes in corneal wavefront aberrations in microincision and small-incision cataract surgery. J Cataract Refract Surg.

[31] Sanders DR. Matched population comparison of the Visian Implantable Collamer Lens and standard LASIK for myopia of -3.00 to -7.88 diopters. J Refract Surg. 2007; 23:537–553. 10.3928/1081-597X-20070601-02 17598571

